# Exercise Delays Human Leukemia Progression and Mitigates Graft-Versus-Host Disease After Donor Lymphocyte Infusion in Xenogeneic Mice

**DOI:** 10.3390/cancers17172826

**Published:** 2025-08-29

**Authors:** Helena Batatinha, Nicole A. Peña, Giovannah A. Hoskin, Timothy M. Kistner, Douglass M. Diak, Grace M. Niemiro, Emmanuel Katsanis, Richard J. Simpson

**Affiliations:** 1 School of Nutritional Sciences and Wellness, The University of Arizona, Tucson, AZ 85724, USA; h.batatinha@uncg.edu (H.B.);; 2 The University of Arizona Cancer Center, The University of Arizona, Tucson, AZ 85724, USA; 3 Department of Immunobiology, The University of Arizona, Tucson, AZ 85724, USA; 4 Department of Pediatrics, The University of Arizona, Tucson, AZ 85724, USA

**Keywords:** exercise-oncology, immunotherapy, tumor metabolism, exercise immunology, voluntary wheel running

## Abstract

**Background:** Donor lymphocyte infusion (DLI) is employed to enhance the graft-versus-leukemia (GvL) effect and improve remission rates following allogeneic hematopoietic cell transplantation (alloHCT). However, graft-versus-host disease (GvHD) remains a significant complication of both alloHCT and DLI. Regular exercise has been shown to reduce cancer risk, enhance treatment responses, and mitigate therapy-related toxicities. This study investigated the effects of voluntary wheel running on GvL and GvHD following DLI in a xenogeneic mouse model. **Methods:** Immunodeficient NSG-IL15 mice were challenged with a luciferase-expressing chronic myelogenous leukemia cell line (K562), and then they received DLI with peripheral blood mononuclear cells (PBMCs) from healthy volunteers (GvL model). Non-tumor bearing mice received DLI to model GvHD. Half of the mice in each group were then given free access to a running wheel. Tumor growth (bioluminescence), GvHD, and body weight were monitored biweekly for ~40 days. **Results:** In the GvHD model, exercise extended overall survival by 60% and reduced GvHD severity. In the GvL model, exercise significantly lowered tumor burden and extended tumor-free survival in both DLI and vehicle control groups by 44.5% and 37.5%, respectively, suggesting both immune-dependent and immune-independent mechanisms. RNA sequencing of bone marrow from saline-injected mice revealed that genes associated with mitochondrial function, protein synthesis, and metabolic processes were downregulated in tumors from exercised mice. **Conclusions:** In summary, voluntary wheel running improved DLI outcomes by enhancing GvL and reducing GvHD. These benefits may be mediated, in part, through exercise-induced metabolic reprogramming of leukemia cells.

## Simple Summary

Donor lymphocyte infusion is a type of immune therapy that helps control leukemia after a stem cell transplant, but it often comes with the serious side effect of graft-versus-host disease, where the donated immune cells attack the patient’s healthy tissues. Exercise has long been known to improve cancer treatment responses and reduce side effects, but its role in this type of therapy has not been well studied. In this research, we tested whether voluntary running could influence the benefits and risks of donor lymphocyte infusion in mice. All mice received leukemia cells, and some were given immune cells from healthy donors. Half of the mice had access to a running wheel, while the others remained sedentary. We found that exercise reduced the severity of graft-versus-host disease, extended survival, and slowed tumor growth. Mice that exercised lived longer and had fewer cancer cells compared to those that did not exercise. Additional analyses suggested that exercise changed how leukemia cells used energy, which may have contributed to these benefits. These findings highlight the potential of exercise to make immune therapies for leukemia more effective and safer for patients.

## 1. Introduction

Leukemia relapse remains the leading cause of mortality following allogeneic hematopoietic cell transplantation (alloHCT). Donor lymphocyte infusion (DLI) is a form of adoptive cellular immunotherapy commonly used after alloHCT to prevent and treat leukemic relapse [1]. The success of DLI is largely attributed to the Graft-vs.-Leukemia (GvL) effect, where the donor-derived lymphocytes can recognize and eliminate residual malignant cells within the recipient. However, this is often associated with graft-versus-host disease (GvHD)—a severe and sometimes fatal complication in which donor lymphocytes attack the host’s healthy tissues, leading to inflammation and tissue damage [2]. Numerous studies have explored depleting selective cell subset in DLI to prevent GvHD. While these approaches may mitigate GvHD, they may also diminish disease control [3].

Regular physical activity is well-documented to reduce cancer risk, improve outcomes in cancer survivors, and serve as an adjuvant therapy for various cancer therapies [4,5]. Each bout of exercise triggers a catecholamine-driven mobilization and redistribution of effector lymphocytes—including natural killer (NK) cells, γδ T cells, and CD8+ T cells—characterized by a cytotoxic and tissue-migratory phenotype [6,7]. The repeated mobilization and circulation of these immune cells are believed to enhance immune surveillance, contributing to reduced cancer risk and slower tumor progression in physically active individuals [8]. Preclinical studies have demonstrated that exercise can enhance NK-cell and CD8+ T-cell infiltration into tumors, thereby suppressing tumor growth in several murine cancer models [9,10,11,12]. Additionally, randomized controlled trials have shown that exercise can mitigate treatment-associated adverse effects, reduce systemic inflammation, and improve quality of life in patients undergoing cancer therapy [13,14,15].

Our group has recently shown that a single bout of exercise performed by the donor could enhance the DLI product and improve outcomes following transfer, in xenogeneic mice. In this study, we investigated whether voluntary wheel running, performed by the recipient, could serve as an adjuvant to DLI therapy by enhancing the GvL response while mitigating GvHD in leukemia-bearing xenogeneic mice. Our findings demonstrated that wheel running not only enhanced DLI efficacy but also reduced tumor burden through an immune-independent mechanism, likely mediated by alterations in tumor metabolism.

## 2. Methods

### 2.1. Participants and Blood Processing

Eleven healthy adults (7 females, 4 males) participated in this study (Mean ± SD: age: 29.5 ± 5 years; BMI: 22.59 ± 1.61 kg/m^2^). Written informed consent was obtained from each volunteer after proper explanation of the procedures and risks. The study was approved by the Human Subjects Protection Program at the University of Arizona (#1801161041). All participants were asked to visit the laboratory between 08:00 and 10:00 local time following an 8–12 h overnight fast (water could be consumed during the fasting period). Blood was collected in acid citrate dextrose (ACD) tubes (Becton-Dickinson, NC, USA). PBMCs were isolated from blood collected in ACD tubes and cryopreserved in liquid nitrogen at a concentration of 10 × 10^6^ cells/mL freezing media (90% FBS, 10% DMSO) until further use in animal experiments.

### 2.2. Cell Lines and Healthy Donor Peripheral Blood Mononuclear Cells (PBMCs)

K562-luc2 cells were thawed from cryopreservation 48 h before tumor injections and cultured at 37 °C with 5% CO_2_ in Iscove’s DMEM supplemented with 10% FBS and 8 µg/mL blasticidin. After two days in culture, the cells were harvested, washed three times with sterile PBS, and resuspended in sterile saline at a concentration of 5 × 10^5^ cells per 200 µL for injection. Cryopreserved PBMCs were thawed in a 37 °C water bath for approximately two minutes, until a small ice pellet remained. The cells were then resuspended in 5 mL RPMI + 10% FBS, supplemented with IL-15 (0.1 mg/mL), and incubated at 37 °C with 5% CO_2_ for one hour to enhance activation and recovery of NK cell function. Following incubation, PBMCs were washed three times with PBS to remove all RPMI media and resuspended in sterile-filtered saline at a concentration of 5 × 10^6^ PBMCs per 200 µL for injection.

### 2.3. Animal Experiments

All experiments were approved by the University of Arizona IACUC (protocol 17-338). Eight- to twelve-week-old NSG-Tg(Hu-IL15) female mice NOD.Cg-Prkdcscid Il2rgtm1Wjl Tg(IL15)1Sz/SzJ (NSG-Tg(Hu-IL15)) (Jackson Labs, Stock No: 030890) were used for xenotransplantation of K562-luc2 tumor cells (ATCC, Manassas, VA, USA) and human PBMCs. These mice express the human IL-15 transgene, enabling physiological levels of human IL-15 expression (7.1 ± 0.3 pg/mL).

For GvHD experiments, mice were irradiated with 100 cGy on Day −1 (Bio5 Cesium 137 Irradiator, Atomic Energy of Canada Ltd., Ottawa, ON, Canada) to enhance human cell engraftment. On Day 0, they received an intravenous injection of 5 × 10^6^ PBMCs (obtained from healthy donors under resting conditions) via the lateral tail vein. The following day (Day +1), half of the mice were placed in cages with free access to a running wheel (80821S Scurry Mouse Activity Wheel—Lafayette Instrument, IN, USA) for the remainder of the experiment. Mice were weighed and assessed for xenogeneic GvHD symptoms following PBMC injections. GvHD scoring was based on the following clinical parameters as described in our previous publication [6]. Mice displaying a combination of severe clinical signs indicative of moribund status were euthanized. Euthanasia (PTS) criteria included a GvHD score ≥ 6 or two consecutive scores ≥ 4; ≥20% body weight loss; or the development of hind limb paralysis or impaired ambulation.

For GvL experiments, mice underwent the same irradiation protocol (100 cGy on Day −1). On Day 0, they were injected intravenously with 5 × 10^5^ K562-luc2 cells via the lateral tail vein. Three days later (Day +3), they received an intravenous injection of 5 × 10^6^ PBMCs or saline (vehicle control). On Day +4, half of the mice in each group were placed in running wheel cages (80821S Scurry Mouse Activity Wheel—Lafayette Instrument, IN, USA). Tumor progression was monitored using bioluminescent imaging (BLI), starting the day after tumor injection, and repeated every 3–4 days (LargoX, Spectral Instruments Imaging, Tucson, AZ, USA). Mice were maintained under 2% isoflurane anesthesia during imaging. Tumor-free survival was calculated based on Day +1 imaging BLI for each mouse. Each PBMC donor sample was injected into 2–3 mice, and all animals were monitored daily. There were 14 mice per group. A total of 42 mice. Mice were co-housed (2 mice per cage) to diminish stress.

Group allocation was performed by an individual who was not involved in data collection or analysis. The person responsible for data collection and analysis was blinded to the group assignments. All mice were euthanized on day +40 via exposure to a lethal dose of CO_2_, following institutional guidelines. Anesthesia was not administered before euthanasia. This work has been reported in line with the ARRIVE guidelines 2.0.

### 2.4. RNA Sequencing

Bone marrow from 10 mice per group was collected and sequenced by bulk RNA-seq (Supplementary Table S1). Sequence reads were demultiplexed using Illumina’s BaseSpace service, San Diego, CA, USA. Adapter sequences and low-quality reads were removed from FastQs using Trimmomatic (v0.39) [16]. Human RNA was parsed using AstraZeneca’s Disambiguate tool [17]. Reads were aligned to the Gencode assembly of the human genome (release 37) using STAR v2.7.3a [18]. Raw expression counts were generated using htseq-count (v0.13.5) [19]. Differential expression analysis was performed using edgeR, Elmira, NY, USA. [20]. Using the output from edgeR, gene set enrichment analysis (GSEA) was done to enrich for Gene Ontology (GO) terms. GSEA was performed using clusterProfiler (v4.10.1), with ranking based on sign of the fold change ×log10 (*p*-value) [21].

### 2.5. Statistical Analysis

Two-way repeated measures analysis of variance with Bonferroni correction for multiple comparisons were used to determine significant longitudinal differences between the main effects (animal groups and time) and the interaction effect (animal group × time) in GvHD scores, and GvL BLI. Simple survival analysis (Kaplan–Meier) was used to determine significance in overall and tumor-free survival. Significance was accepted at *p* < 0.05. For GSEA, FDR < 0.05 was used to correct for multiple hypothesis testing.

## 3. Results

### 3.1. Voluntary Wheel Running Reduces Xenogeneic GvHD and Extends Survival After DLI in NSG-IL15 Mice

**Figure 1:**
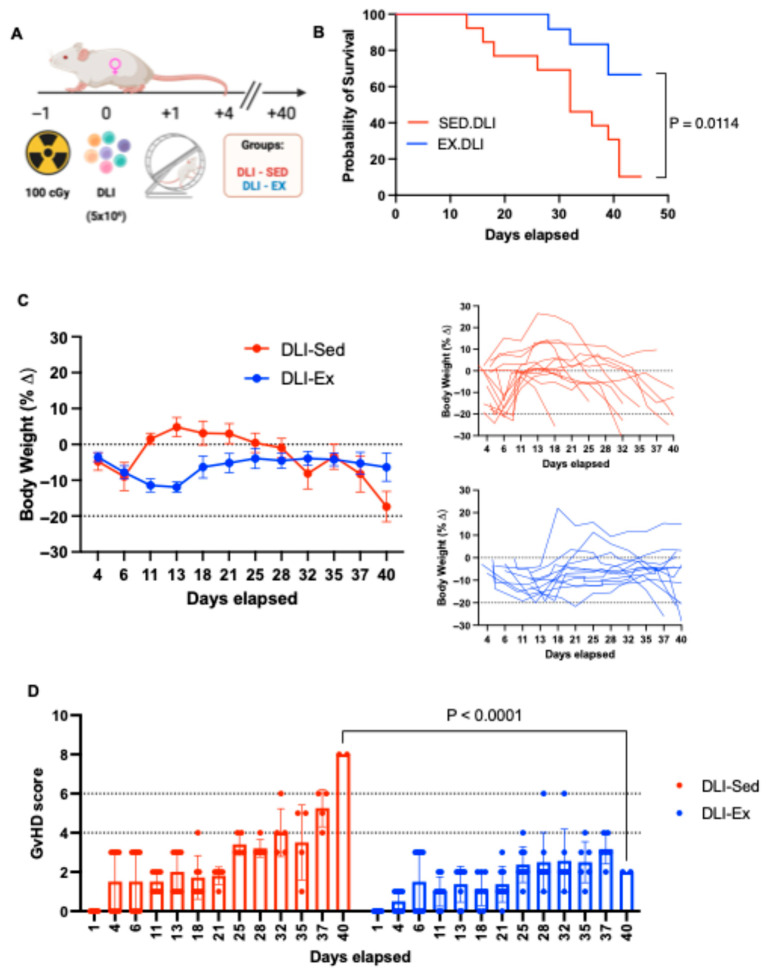
Exercise extends survival and reduces GvHD. (**A**) Illustration of the experimental design. (**B**) Kaplan–Meier analysis of overall survival. (**C**) Body weight measured bi-weekly, presented as mean ± SE along with individual data points (Spaghetti plots). (**D**) Xenogeneic GvHD scores measured bi-weekly presented as mean ± SE. Groups include: DLI-treated sedentary mice (Red line), and DLI-treated mice with access to a running wheel (blue line). *p*-values are shown on the graphs.

To assess the impact of exercise on xenogeneic GvHD (xGvHD) following DLI, NSG-IL15 mice were infused with PBMCs from healthy human donors and either remained sedentary in standard cages (DLI-Sed) or were given free access to a running wheel (DLI-Ex). Body weight was monitored and xGvHD severity was scored twice a week until day +40 (Figure 1A). Throughout the experiment, all mice with access to a running wheel averaged 5 km daily. Mice were co-housed (2 mice per cage) to reduce stress. Given that, individual running data was not measured. By the study’s end, 70% of the exercising mice remained alive, compared to only 10% in the sedentary group (Figure 1B). The DLI-Ex group initially lost weight but maintained a stable body weight throughout the study. In contrast, DLI-Sed mice initially gained weight followed by rapid weight loss due to xGvHD during the course of the experiment, with some requiring euthanasia (Figure 1C). Similarly GvHD scores were consistently higher in the sedentary group, reaching statistical significance by day 40 (*p* < 0.0001) (Figure 1D). These findings suggest that voluntary exercise improves survival and provides protection against xGvHD following DLI.

### 3.2. Exercise Enhances the GvL Effects of DLI and Provides Additional Immune-Independent Leukemia Control

**Figure 2:**
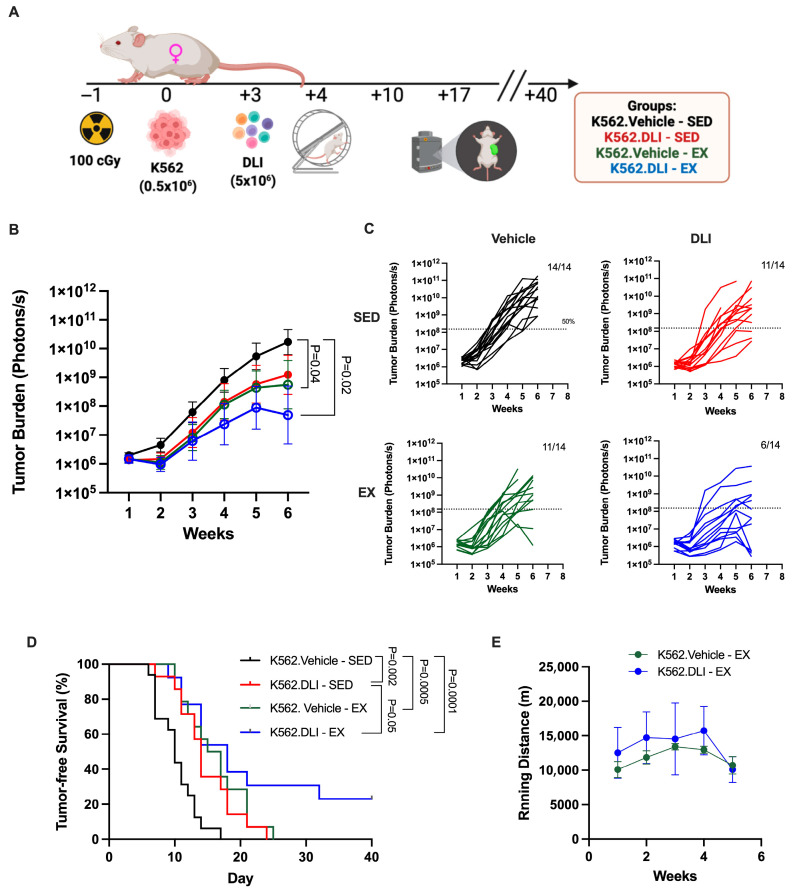
Exercise reduces tumor burden and delays leukemia progression. (**A**) Schematic of the experimental design. (**B**) Tumor burden measured weekly by bioluminescence imaging (BLI), presented as mean photon flux (photons/s) (**C**) with individual data points. Groups include: vehicle-treated sedentary mice (black line), DLI-treated sedentary mice (red line), vehicle-treated mice with access to a running wheel (green line), and DLI-treated mice with access to a running wheel (blue line). (**D**) Kaplan–Meier analysis of tumor-free survival. Mice were considered tumor-free until their BLI value exceeded the individual baseline measured on day 1. (**E**) Average of daily running distance per cage, in each week. *p*-values are shown on the graphs.

To evaluate whether exercise enhances the GvL effects of DLI, leukemia-bearing NSG-IL15 mice were infused with PBMCs three days after leukemia challenge with the human chronic myeloid leukemia cell line, K562. Mice were either given access to a running wheel or kept in standard cages without exercise (Figure 2A). The combination of DLI and exercise resulted in reduced tumor progression (Figure 2B,C), and extended tumor-free survival (Figure 2D) compared to both the vehicle control and the sedentary DLI group. Given that mice were co-housed to diminish stress, individual running data were not collected; however, the average daily running per cage is shown in Figure 2E. No statistical differences were seen for groups or time. Interestingly, mice with access to a running wheel that were challenged with tumor but did not receive DLI, also exhibited decreased tumor burden and prolonged tumor-free survival compared to their sedentary counterparts. This finding suggests that exercise-mediated tumor control extends beyond immunological mechanisms, as these mice lack functional immune cells and received vehicle instead of human PBMCs.

### 3.3. Voluntary Exercise Downregulates Leukemia Metabolism Genes

**Figure 3:**
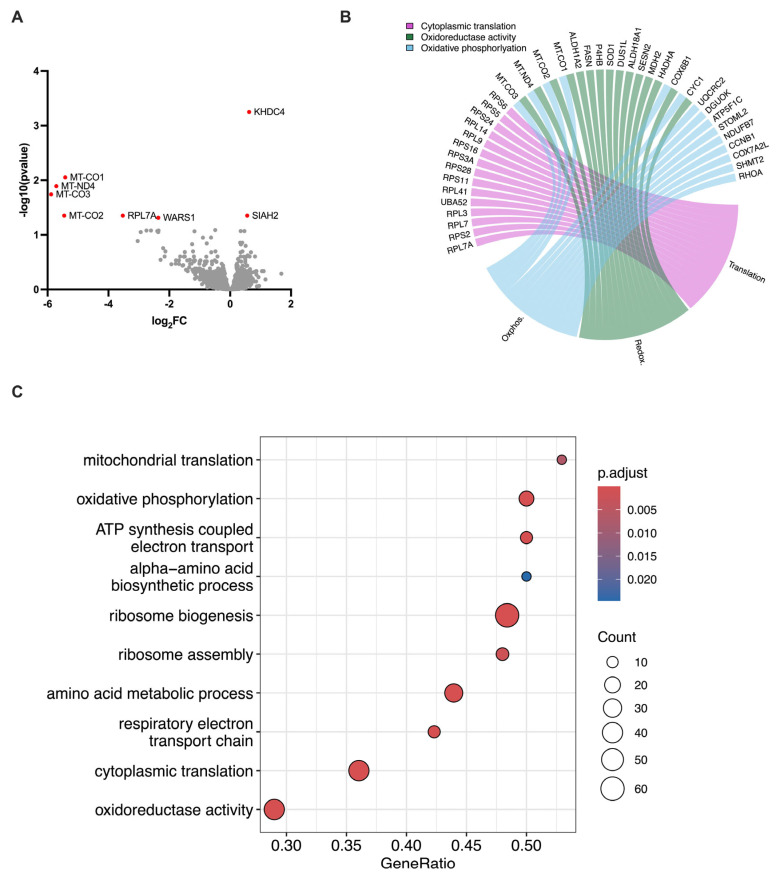
(**A**) Volcano plot showing differentially expressed genes comparing tumors in mice with access to a wheel versus those in sedentary cages. Genes that are significantly differentially expressed are named and highlighted in red. A negative log_2_FC represents genes that are downregulated in exercising animals when compared to sedentary animals. (**B**) Chord diagram representing the 15 most differentially expressed genes that are driving enrichment in a given GO term. (**C**) Dotplot showing selected Gene Ontology (GO) pathways from gene set enrichment analyses (GSEA). Count refers to the number of core genes in our dataset that drive enrichment of a specific GO term and gene ratio is the number of core enrichment genes divided by the count of pathway genes that are present in our ranked dataset.

To further investigate the DLI-independent effects of exercise on leukemia control, we collected bone marrow on day 40 from wheel-running and sedentary mice that received no DLI and performed bulk RNA sequencing to analyze human tumor cells in the mouse bone marrow. We found that exercise downregulated many human genes, primarily those associated with mitochondrial function (e.g., MT-CO1, MT-ND4) and protein synthesis (e.g., RPL7A, WARS1) (Figure 3A). Gene set enrichment analysis (GSEA) revealed downregulation of pathways involved in metabolic processes, including aerobic and anaerobic respiration, protein synthesis, oxidative phosphorylation, mitochondrial function, and energy metabolism (Figure 3B,C). Altogether, these findings suggest that exercise might reduce leukemia progression by impairing tumor metabolism and protein synthesis, potentially limiting cell proliferation and making the tumor more sensitive to the GvL effects of DLI.

## 4. Discussion

This is the first study to investigate the impact of exercise following DLI on both GvL and GvHD outcomes in a xenogeneic mice model. Our findings demonstrate that exercise can function as valuable adjuvant in the treatment of leukemia. Mice that engaged in voluntary wheel running exhibited reduced xGvHD severity and delayed tumor progression following DLI compared to sedentary controls receiving the same immunotherapy. Additionally, we observed that the anti-leukemic effects of exercise were associated with decreased tumor cell metabolic activity, independently of DLI. This exploratory study provides new insights into strategies to improve leukemia outcomes and highlights potential mechanisms by which exercise may modulate tumor biology.

GvHD is a major cause of treatment-related morbidity and mortality following DLI [22]. High-dose corticosteroids and immunosuppressants are the first-line therapies for GvHD. Yet, their high failure rate—approximately 40%—and poor treatment outcomes remain significant challenges. Here, we have shown that incorporating physical exercise after DLI extended survival by 60% and reduced xGvHD clinical scores in mice. This suggests that exercise may reduce DLI-associated GvHD toxicity and potentially enhance its anti-leukemia efficacy.

Prior studies have also demonstrated the beneficial effects of exercise in murine models of GvHD. Fiuza-Luces and colleagues conducted 11 weeks of moderate-intensity treadmill training in two murine models: one MHC-matched to assess acute GvHD, and one miHA-mismatched to assess chronic GvHD. All mice received total body irradiation before transplantation. In both models, exercise improved physical capacity following allo-HCT, which was associated with a reduction in total GvHD clinical scores. However, no statistically significant differences in overall survival were observed [23]. In a subsequent study, the same group found that combining exercise training with standard immunosuppressive therapy led to extended survival, lower GvHD clinical scores, and reduced inflammation in the same murine model [24]. Collectively, these findings support the potential of exercise as an adjunct therapy to reduce GvHD severity.

The mechanisms through which exercising the recipient enhances the anti-leukemic activity of DLI remain to be fully defined. One plausible explanation is that exercise-induced mobilization and redistribution of effector lymphocytes increased the likelihood of contact between DLI-derived immune cells and leukemic targets, thereby promoting leukemia clearance [8,12]. Importantly, we also observed an exercise-induced suppression of leukemia burden in the absence of DLI, indicating a potential immune-independent effect. This tumor-intrinsic modulation may have rendered leukemic cells more susceptible to subsequent GvL responses by CD8+ T-cells and NK-cells. Consistent with this, RNA sequencing of human leukemic cells isolated from the bone marrow of exercised mice revealed metabolic reprogramming that occurred independently of DLI and may underlie this enhanced sensitivity. Several studies have investigated tumor metabolism in the context of tumor development and progression [25,26,27,28]. Most tumor cells rely on glycolysis and anaerobic metabolism to produce ATP, including leukemia cells [29]. However, mitochondrial metabolism has also been shown to be critical for the progression of myeloid malignancies [29], and is currently being explored as a potential therapeutic target. Beauchamp and colleagues provide an in-depth review of how OXPHOS is required for leukemia progression and is associated with treatment resistance [30]. Here, we observed that exercise downregulated mitochondrial genes and gene sets related to OXPHOS and ATP synthesis in bone marrow samples from exercised chronic myeloid leukemia (CML) xenogeneic mice.

Kuntz and collaborators [31] demonstrated that OXPHOS is upregulated in early CML cells, correlating with increased proliferation and survival. Notably, the combination of imatinib—a tyrosine kinase inhibitor (TKI)—and tigecycline—an antibiotic that disrupts mitochondrial function—effectively eradicated early CML in a xenogeneic model. This effect was not observed with imatinib alone. Giustacchini et al. [32] performed scRNAseq on stem cells collected from CML patients and found that malignant stem cells upregulated genes associated with OXPHOS compared to non-malignant stem cells from the same individuals. Furthermore, downregulation of OXPHOS gene expression was associated with a favorable response to TKI therapy, further supporting the role of mitochondrial metabolism in early CML proliferation and treatment resistance. In our model, exercise was initiated four days after tumor challenge. Given the evidence that OXPHOS is essential in the initial stages of CML development, the early introduction of voluntary running may have contributed to improved tumor control. Moreover, the lowest tumor burden was observed when exercise was combined with DLI treatment, suggesting that exercise-induced mitochondrial dysfunction of tumors may enhance DLI efficacy.

While this study provides important new insights, important limitations should be noted. First, we did not test the effects of exercise in combination with standard leukemia therapies such as chemotherapy or antibody-based treatments. Since donor lymphocyte infusion is often used as a stand-alone therapy, our focus here was to isolate the contribution of exercise, but future work will be needed to examine exercise within combination treatment strategies. Second, because mice were housed together, we were unable to measure individual running activity, which limits our ability to determine how the amount of exercise performed by each mouse influenced outcomes. However, our findings demonstrate that voluntary exercise can improve both anti-leukemia responses and reduce graft-versus-host disease following donor lymphocyte infusion, supporting further investigation in more clinically relevant settings.

## 5. Conclusions

In conclusion, voluntary exercise improves overall survival, reduces xGvHD, and delays tumor progression in immunocompromised mice. These benefits are accompanied by a downregulation of mitochondrial metabolism in leukemia cells, suggesting a potential mechanism by which exercise modulates tumor biology. While further research is needed to fully elucidate the pathways involved, our results highlight the potential of exercise as a non-pharmacological adjuvant to improve immunotherapy outcomes in leukemia.

## Data Availability

Sequencing data will be made publicly available at Gene Expression Omnibus (GEO). The raw data is included in the supplemental material. The corresponding author can be contacted for any other data requests.

## Supplementary Materials

The following supporting information can be downloaded at: https://www.mdpi.com/article/10.3390/cancers17172826/s1; Table S1: RNAseq data.
